# Association Between Night-Shift Work and Cancer Risk: Updated Systematic Review and Meta-Analysis

**DOI:** 10.3389/fonc.2020.01006

**Published:** 2020-06-23

**Authors:** Aishe Dun, Xuan Zhao, Xu Jin, Tao Wei, Xiang Gao, Youxin Wang, Haifeng Hou

**Affiliations:** ^1^School of Basic Medical Science, Shandong First Medical University & Shandong Academy of Medical Sciences, Tai'an, China; ^2^School of Public Health, Shandong First Medical University & Shandong Academy of Medical Sciences, Tai'an, China; ^3^Department of Otolaryngology, Head and Neck Surgery, Beijing Tongren Hospital, Capital Medical University, Beijing, China; ^4^Beijing Key Laboratory of Clinical Epidemiology, School of Public Health, Capital Medical University, Beijing, China

**Keywords:** night-shift work, carcinogenicity, meta-analysis, risk factor, odds ratio

## Abstract

**Background:** Nightshift work introduces light at night and causes circadian rhythm among night workers, who are considered to be at increased risk of cancer. However, in the last 2 years, nine population-based studies reported insignificant associations between night-shift work and cancer risks. We aimed to conduct an updated systematic review and meta-analysis to ascertain the effect of night-shift work on the incidence of cancers.

**Methods:** Our protocol was registered in PROSPERO and complied with the Preferred Reporting Items for Systematic Reviews and Meta-Analyses (PRISMA). Embase, PubMed, and Web of Science databases were used to comprehensively search studies published up to May 31, 2019. The random-effect model (Der Simonian-Laird method) was carried out to combine the risk estimates of night-shift work for cancers. The dose-response meta-analysis was performed to verify whether the association was in a dose-dependent manner.

**Results:** Our literature searching retrieved 1,660 publications. Included in the meta-analyses were 57 eligible studies with 8,477,849 participants (mean age 55 years; 2,560,886 men, 4,220,154 women, and 1,696,809 not mentioned). The pooled results showed that night-shift work was not associated with the risk of breast cancer (*OR* = 1.009, 95% CI = 0.984–1.033), prostate cancer (*OR* = 1.027, 95% CI = 0.982–1.071), ovarian cancer (*OR* = 1.027, 95% CI = 0.942–1.113), pancreatic cancer (*OR* = 1.007, 95% CI = 0.910–1.104), colorectal cancer (*OR* = 1.016, 95% CI = 0.964–1.068), non-Hodgkin's lymph (*OR* = 1.046, 95% CI = 0.994–1.098), and stomach cancer (*OR* = 1.064, 95% CI = 0.971–1.157), while night-shift work was associated with a reduction of lung cancer (*OR* = 0.949, 95% CI = 0.903–0.996), and skin cancer (*OR* = 0.916, 95% CI = 0.879–0.953). The dose-response meta-analysis found that cancer risk was not significantly elevated with the increased light exposure of night- shift work.

**Conclusion:** This systematic review of 57 observational studies did not find an overall association between ever-exposure to night-shift work and the risk of breast, prostate ovarian, pancreatic, colorectal, non-Hodgkin's lymph, and stomach cancers.

## Introduction

Night-shift work is increasingly frequent among both full-time and part-time workers worldwide. Night-shift workers have to face the biological challenges of work shifts, light at night and altered circadian rhythm cycles. These challenges, as well as alterations in daily life and activity may introduce potential harms to night workers. In different employment sectors today, the number of people working overtime or on a night shift has been increasing, especially in transportation, health care, and manufacturing (1). Surveys of Americans, Europeans and Australians have shown that 15–30% of adults were engaged in shift work experience, and more than 30% of them fell asleep at work at least once a week (2). Apart from an increased risk of work-related injury, night-shift workers have a greater chance of having long-term disorders. Currently, epidemiological evidences indicated that night-shift work is recognized to be associated with increased susceptibilities to cancer (3, 4).

The International Agency for Research on Cancer (IARC) has defined that night-shift work is probably carcinogenic to humans (IARC Group 2A) (5). Further studies have proposed the followings as a potential mechanism of carcinogenicity of night-shift work as: (1) circadian rhythm disruption, (2) melatonin suppression due to exposure to light at night, (3) physiological changes, (4) lifestyle disturbances, and (5) decreased vitamin D levels (resulting from lack of sunlight) (6). However, studies focusing on the association between night-shift work and cancer risks have reached contradictory conclusions. Even though several systematic reviews and meta-analyses have been conducted, they presented inconsistent findings (7). Nine reviews reported that night work may be positively associated with breast cancer, skin cancer, prostate cancer, colorectal cancer, and lung cancer (2, 8–15), while four reviews reported slightly elevated but statistically insignificant results, among which the review published in 2017 included nine studies and 2,570,790 participants (2), the review in 2016 included ten studies and 4,660 breast cancer patients (16), the review in 2013 included 16 studies and 1,444,881 participants (17), and another review in 2013 included 15 studies and 1,422,189 participants (18).

In the last 2 years, nine original population-based studies reported that night-shift work was not associated with cancer development, which have not been included in previously published reviews (19–26). We conducted this study to systematically summarize the evidence regarding the associations between night-shift work and cancer risks. We expect to facilitate recognition of the health-related problems among night-shift workers.

## Materials and Methods

This systematic review and meta-analysis were conducted in accordance with the Preferred Reporting Items for Systematic Reviews and Meta-Analyses (PRISMA) (Table S1). The study protocol was registered in the online database of PROSPERO (CRD42019138215). This systematic review aimed to answer the medical question of the association between night-shift work and cancer risks by reference to PICOS: (1) study population were night-shift workers; (2) compared to population without night-shift work; (3) the exposure was defined as night-shift work; (4) the outcome of cancer risk was evaluated; (5) observational studies on this topic were included.

### Search Strategy

We used Embase, PubMed, and Web of Science databases to systematically search English language publications issued up to May 31, 2019. The search terms were “carcinoma” or “tumor” or “cancer” or “neoplasm,” and “night-shift work” or “night work” or “shift work” or “work schedule tolerance” or “rotating-shift work.” The detailed literature search strategy was shown in Supplementary Box 1. Two investigators independently searched and then screened the retrieved studies. In addition, we manually screened the reference lists of included studies to collect additional literature.

### Inclusion and Exclusion Criteria

Literature was included based the following criteria: (1) night-shift work was reported. Night-shift work was defined by questionnaire interview or occupational history of those who have ever exposed to shift system (rotating or fixed, forward or backward rotation). The durations that participants have ever engaged in night-shift work were collected by retrospective investigation or follow-up interview. (2) Cancer risk was investigated. (3) Cohort studies, case-control studies, or nested case-control studies. (4) The risk was estimated by odds ratio (OR), risk ratio (RR), or hazard ratio (HR), with 95% confidence interval (CI). (5) For studies reporting overlapping data, the studies newly published or with a larger sample size were included. (6) Publications in English language. Exclusion criteria were (1) studies without sufficient data; (2) studies referring to recurrent cancer.

### Quality Assessment

We assessed the bias risk as low, high, or unclear by verifying the checklist for measuring bias in risk factor studies to counter 10 important sources (domains) of bias (17, 27). The following are domains of bias risk assessment: (1) exposure definition, (2) exposure assessments, (3) reliability of assessments, (4) analysis methods in research (research-specific bias), (5) confusion, (6) attrition, (7) blinding of assessors, (8) selective reporting, (9) funding, and (10) conflict of interest. We then rated the study-level risk of bias as: low (low risk in all major domains and ≥2 of the minor domains), moderate (low risk of bias in ≥4 major and 2 minor domains), or high risk of bias (low risk of bias in <4 major domains). The detailed information is available in Supplementary Box 2.

### Data Extraction

The following items were extracted from eligible studies: (1) first author; (2) publication year; (3) country of participants; (4) study design (cohort studies, case-control studies, or nested case-control studies); (5) number of participants (6) number of cases; (7) duration or person-years of follow-up (8) characteristics of participants (e.g., age, sex, and occupation); (9) years of night- shift work, (10) types of night-shift work; (11) adjusted effect estimates (i.e., OR, RR, and HR) with 95% CI; (12) types of cancers; (13) adjusted variables. Two investigators independently undertook data extraction, and the third author participated in handling debatable issues if necessary.

### Statistical Analysis

All statistical analyses were done with the Stata14.0 software (Stata Corp, College Station, TX, USA). We preferentially measured the association between night-shift work and all cancer risks *via* the pooled estimates (i.e., OR, RR, and HR) and 95% CI. The Q test along with *I*^2^ statistic was used to identify whether heterogeneity was significant between eligible studies. When *P* < 0.10 or *I*^2^ > 50% heterogeneity was considered significant, therefore the random-effect model (Der Simonian-Laird method) meta-analysis was applied, otherwise, the fixed-effect model was used. In addition, subgroup analyses were conducted to stratify the results on specific study design, occupation, night-shift work status, cancer type, and sex. A dose-response meta-analysis was performed to evaluate the risk for cancers per year increase of night-shift work. We established the curve of dose-response relationship using a method proposed by Greenland and Longnecker (28). In this process, we combined the data from studies that reported the estimate effects of cancer risks with night-shift work at ≥3 quantitative categories. To assess the stability of the results, sensitivity analysis was conducted by sequential removal of each original study. Potential publication bias was assessed by the Begg's regression asymmetry test and funnel plot.

## Results

### Literature Search and Study Selection

**Figure 1:**
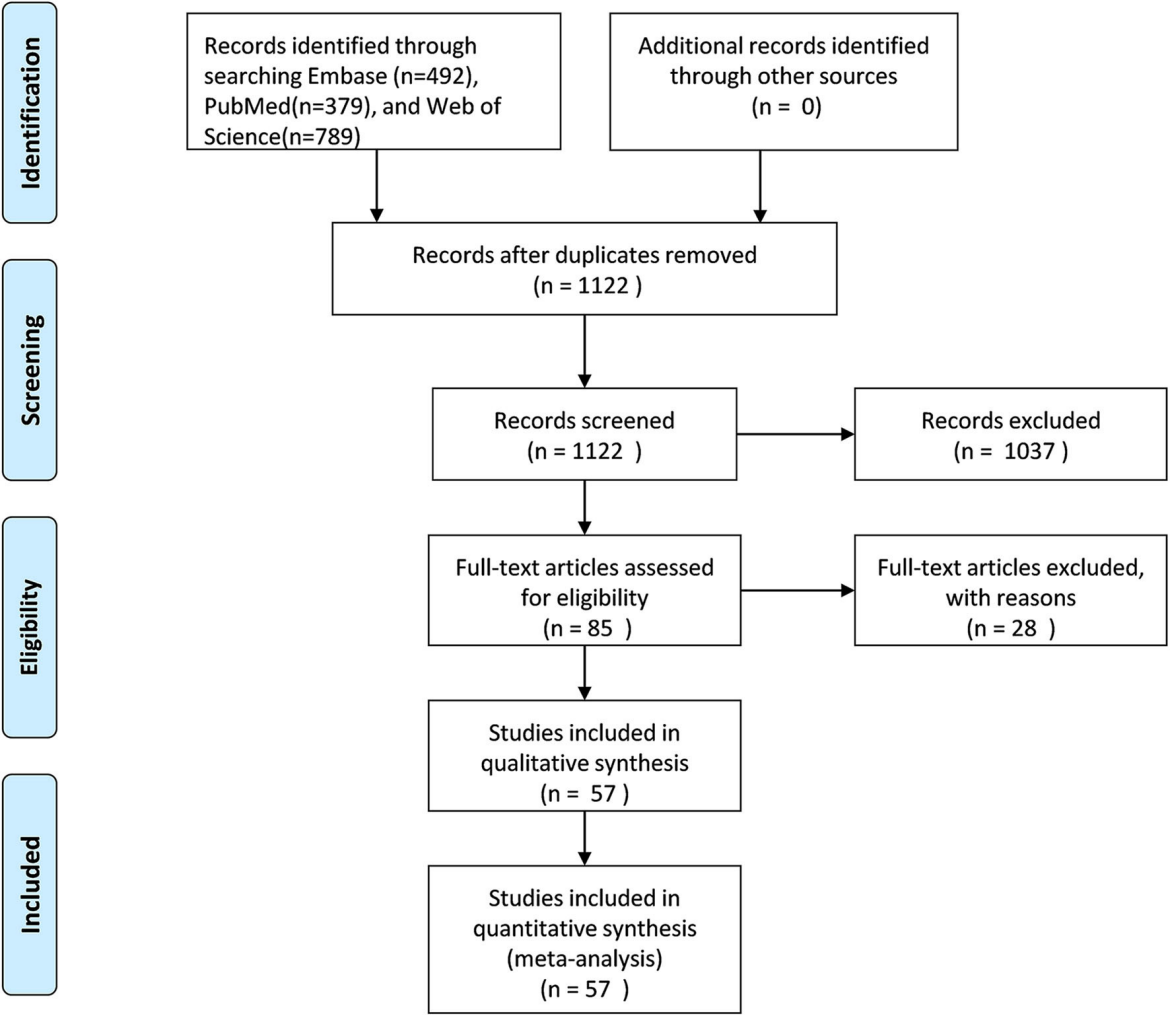
Flow graph of study search and screening.

Details of the literature search and screening are shown in Figure 1. A total of 1660 publications were initially retrieved from Embase, PubMed and Web of Science. Among them, 538 duplicate publications were removed. After review of abstracts 1,037 studies were excluded for the following reasons: not human studies (*n* = 53), not studies on cancer and night-shift work (*n* = 713), reviews/editorials/letters (*n* = 271). By full-text review eight studies with overlapping data and 20 studies on sleep patterns were removed. Altogether, this meta-analysis included 57 articles.

### Characteristics of Included Studies

**Figure 2:**
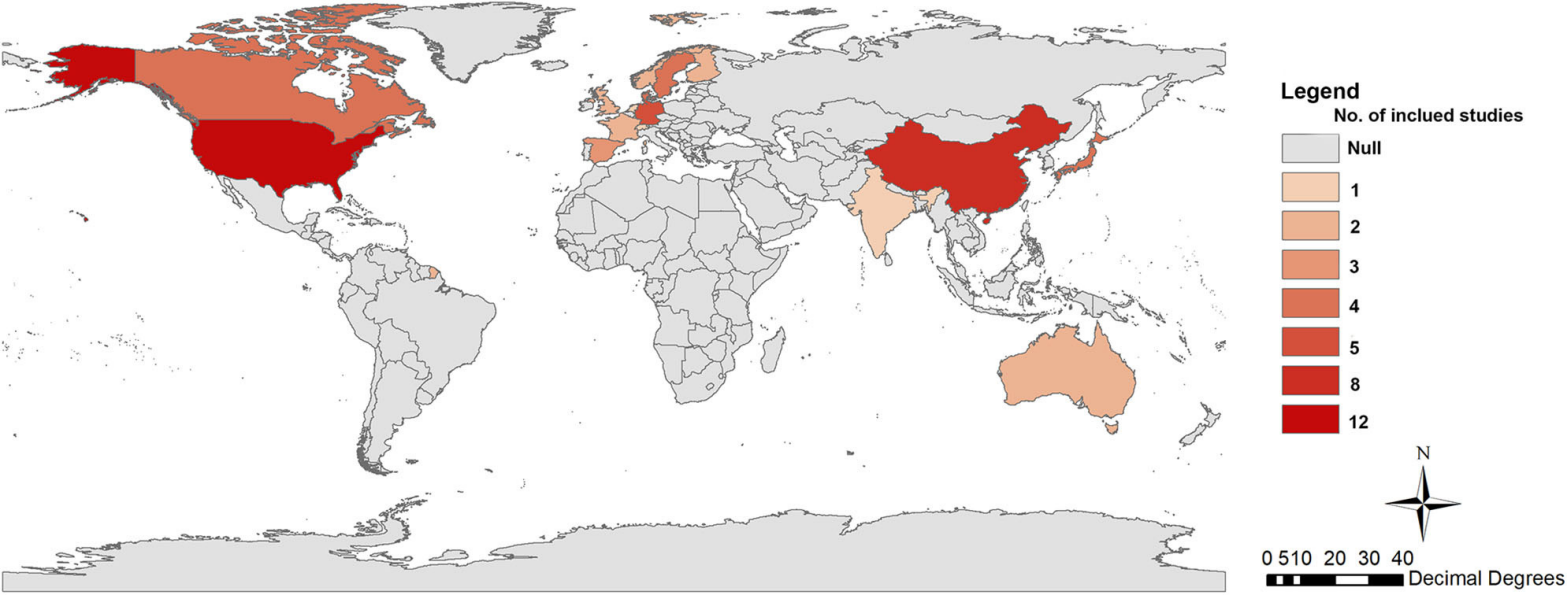
Geographic distribution of included studies.

As shown in Table 1, our study included 57 articles with 8,477,849 participants (mean age 55 years; 2,560,886 men, 4,220,154 women, and 1,696,809 sex not mentioned) (8, 16, 19–26, 29–75). Of these, 13 studies were from Asia, 26 were from Europe, 16 were from North America, and 2 were from Oceania. The geographic distribution of the studies included are shown in Figure 2. In terms of the participants' occupations, 11 studies were conducted among nurses, and two were among textile workers. These studies investigated the association between night-shift work and the risk of cancer in the breast (*n* = 26 studies), prostate (*n* = 12), ovaries (*n* = 8), pancreas (*n* = 6), colon/rectum (*n* = 6), lung (*n* = 6), stomach (*n* = 4), skin (*n* = 4), urinary tract (*n* = 3), esophagus (*n* = 3), uterus (*n* = 2), oral cavity (*n* = 2), larynx (*n* = 2), and testes (*n* = 2), as well as non-Hodgkin's lymphoma (*n* = 5), and leukemia (*n* = 3).

**Table 1:** Characteristics of included studies.

**References**	**Country**	**Study design**	***N* of participants**	**Mean age**	***N* of case**	**Occupations**	**Estimates of risk**	**Cancer**	**Covariates adjusted**	**Measurement of night-shift work**
Hansen et al. (29)	Denmark	CC	12,485	NA	6,281	NA	OR = 1.5^Δ^^▴^	Breast cancer	Age, social class, age at birth of first child, age at birth of last child, and number of children	Interview
Davis et al. (30)	US	CC	1,606	NA	767	NA	OR = 1.13^Δ^^▴^	Breast cancer	Parity, family history of breast cancer, oral contraceptive use, and recent is continued use of hormone replacement therapy	Interview
Lie et al. (31)	Norway	NCC	44,835	NA	537	Nurses	0–14 y: OR = 0.95^Δ^^▴^ 15–29 y: OR = 1.29^Δ^^▴^ ≥30 y: OR = 2.21^Δ^^▴^	Breast cancer	Total duration of work as a nurse and parity	Self-report
Kubo et al. (32)	Japan	CS	14,052	52.14	31	NA	Fixed NSW: RR = 2.3^Δ^^▴^ Rotating NSW: RR = 3.0^Δ^^▴^	Prostatic cancer	Age, study area, family history of prostate cancer, body mass index, smoking, alcohol drinking, job type, physical activity at work, workplace, perceived stress, educational level, and marriage status	Questionnaire
O'Leary et al. (33)	US	CC	1,161	57.19	835	NA	OR = 1.04^Δ^^▴^	Breast cancer	Age at reference date, parity, family history, education, and history of benign breast disease.	In-house interview
Schwartzbaum et al. (34)	Sweden	CS	3,250,787	NA	300,771	NA	Female: OR = 1.00^Δ^^▴^ Male: OR = 1.02^Δ^^▴^	All cancer	Age, socioeconomic status, occupational position, and county of residence of residence	Personal interviews
Viswanathan et al. (4)	US	CS	53,487	53.51	515	Nurses	0–9 y: RR = 0.89^Δ^^▴^ 10–19 y: RR = 1.06^Δ^^▴^ ≥20 y: RR = 1.47^Δ^^▴^	Uterus cancer	Age, age at menarche, age at menopause, parity, BMI, oral contraceptive use, use and duration of postmenopausal hormones, hypertension, diabetes, and smoking	Questionnaire
Marino et al. (35)	US	CC	2,125	NA	812	NA	OR = 1.2^Δ^^▴^	Ovarian cancer	Multivariable adjustment	Interviews
Lahti et al. (36)	Finland	CS	1,669,272	NA	3,813	NA	RR = 1.10^Δ^^▴^	Non-Hodgkin's lymphoma	Age, social class, and cohort period	Questionnaire
Pronk et al. (37)	China	CS	73,049	52.50	717	NA	0–14 y: HR = 1.1^Δ^^▴^ 15–25 y: HR = 0.9^Δ^^▴^ >25 y: HR = 1.0^Δ^^▴^	Breast cancer	Age, education, family history of breast cancer, number of pregnancies, age at first birth, and occupational physical activity	Interview
Chu et al. (38)	China	NCC	2,023	NA	408	NA	HR = 2.54^Δ^^▴^	Breast cancer	Potential cofounders	Interview
Pesch et al. (39)	Germany	CC	1,749	NA	857	NA	>0–4 y: OR = 0.64^Δ^^▴^ 5–9 y: OR = 0.93^Δ^^▴^ 10–19 y: OR = 0.91^Δ^^▴^ ≥20 y: OR = 2.49^Δ^^▴^	Breast cancer	A potential selection bias using bootstrapping, family history of breast cancer, hormone replacement use, and number of mammograms	Interview
Poole et al. (40)	US	CS	181,548	57.21	718	Nurses	1–2 y: HR = 1.07^Δ^^▴^ 3–5 y: HR = 0.90^Δ^^▴^ 6–9 y: HR = 0.92^Δ^^▴^ 10–14 y: HR = 1.14^Δ^^▴^ 15–19 y: HR = 1.28^Δ^^▴^ ≥20 y: HR = 0.80^Δ^^▴^	Ovarian cancer	Age, duration of oral contraceptive use, parity, BMI, smoking status, tubal ligation history, menopausal status, family history of ovarian cancer, duration of breast treating, and cohort	Questionnaire
Kubo et al. (41)	Japan	CS	4,995	55.5	17	NA	RR = 1.79^Δ^^▴^	Prostate cancer	Age, body mass index, alcohol intake, smoking, exercise, and marital status	Questionnaire
Lie et al. (26)	Norway	NCC	1,594	54.46	699	Nurses	1–14 y: OR = 1.2^Δ^^▴^ 15–29 y: OR = 1.2^Δ^^▴^ ≥30 y: OR = 0.8^Δ^^▴^	Breast cancer	Age, period of diagnosis, parity, family history of breast cancer in mother or sister (no/yes), and frequency of alcohol consumption at time of diagnosis	Telephone interview
Hansen et al. (29)	Denmark	CS	1,117	NA	141	Women military	OR = 1.4^Δ^^▴^	Breast cancer	Age, hormone replacement therapy, number of childbirths, age at menarche, years of education, occasional sunbathing frequency, tobacco smoking status	Questionnaire
Parent et al. (42)	Canada	CC	3,670	59.18	761	NA	<5 y: OR = 1.93^Δ^^▴^ 5–10 y: OR = 1.51^Δ^^▴^ >10 y: OR = 1.67^Δ^^▴^	Lung cancer	None	Interview
Natti et al. (43)	Finland	CS	3,095	36.68	51	NA	Men: HR = 1.78^Δ^^▴^ Women: HR = 2.82^Δ^^▴^	All cancer	Age, and smoking status, and health- and work-related factors	Interview
Lin et al. (44)	Japan	CS	22,224	52.19	16	Industry	Fixed NSW: RR = 0.61^Δ^^▴^ Rotating NSW: RR = 0.83^Δ^^▴^	Pancreatic cancer	Age, body mass index, history of diabetes, alcohol drinking, cigarette smoking, perceived stress, and sleep time.	Questionnaire
Knutsson et al. (45)	Sweden	CS	4,036	42.31	94	NA	HR = 2.02^Δ^^▴^	Breast cancer	Number of children, alcohol consumption, BMI, height, weight, waist, hip circumference, educational level, smoking menopausal status, status of oral contraceptive use, and hormones other than contraceptives	Questionnaire
Bhatti et al. (46)	US	CC	3,322	NA	1,490	NA	Invasive: OR = 1.24^Δ^ Borderline: OR = 1.48^Δ^	Ovarian cancer	Age at reference, county, reference year, duration of oral contraceptive use, number of full-term pregnancies, and BMI at age 30	Interviews self-reported
Fritschi et al. (47)	Australian	CC	2,987	NA	1,202	NA	OR = 1.16^Δ^^▴^	Breast cancer	Night shift work	Questionnaire
Menegaux et al. (48)	France	CC	2,549	NA	1,232	NA	OR = 1.27^Δ^^▴^	Breast cancer	Age, study area, parity, age at first full term pregnancy, age at menarche, family history of breast cancer, current hormonal replacement therapy, BMI, tobacco, and alcohol	Interview
Grundy et al. (49)	Canada	CC	2,313	57.03	1,134	NA	0–14 y: OR = 0.95^Δ^^▴^ 15–29 y: OR = 0.93^Δ^^▴^ ≥30 y: OR = 2.21^Δ^^▴^	Breast cancer	Years of night shift history	Questionnaire
Rabstein et al. (50)	Germany	CC	1,749	NA	857	NA	OR = 1.01^Δ^^▴^	Breast cancer	Family history of breast cancer, hormone replacement use, number of mammograms, and estrogen receptor status	Interview
Koppes et al. (51)	Netherland	CS	285,723	NA	2,531	Employed women	HR = 0.87^Δ^^▴^	Breast cancer	Age, origin, children in household education, occupation, job tenure (years)	Interview
Gapstur et al. (52)	US	CS	305,057	51.44	4,836	NA	Fixed NSW: RR = 0.72^Δ^^▴^ Rotating NSW: RR = 1.08^Δ^^▴^	Prostate cancer	Age, race, education, BMI, smoking status, family history of prostate cancer, and painful/frequent urination	Questionnaire
Carter et al. (53)	US	CS	161,004	50.28	1,253	Employed women	Fixed NSW: R = 1.12^Δ^^▴^ Rotating NSW: RR = 1.27^Δ^^▴^	Ovarian cancer	Oral contraceptive use, age at menarche and menopause, tubal ligation, parity, postmenopausal estrogen use, race, family history of breast/ovarian cancers, exercise, BMI, and height	Questionnaire
Yong et al. (54)	Germany	CS	27,828	40.05	1,073	Chemical workers	HR = 1.04^Δ^	All cancer	Age, job level, cigarette smoking, and employment duration in categories	Questionnaire
Ren et al. (55)	China	CC	1,454	NA	712	NA	OR = 1.34^Δ^^▴^	Breast cancer	Age, education, BMI, marital status, age at menarche, menopausal status, parity, activity, breastfeeding, family history of breast cancer, and other sleep factors	Database
Datta et al. (56)	India	CC	150	NA	50	NA	OR = 1.51^Δ^^▴^	Breast cancer	Age, obesity factors, and food habits	Interview
Kwon et al. (57)	China	CS	4,471	54.01	1,451	Textile workers	0–17.1 y: HR = 0.76^Δ^^▴^ 17.1–24.9 y: HR = 0.89^Δ^^▴^ 24.9–30.6 y: HR = 0.94^Δ^^▴^ >30.6 y: HR = 0.82^Δ^^▴^	Lung cancer	Age, smoking, parity, and endotoxin	Factory record
Gu et al. (58)	US	CS	71,857	63.98	5,413	Nurses	1-5 y: HR = 1.03^Δ^^▴^ 6–14 y: HR=1.04^Δ^^▴^ ≥15 y: HR = 1.08^Δ^^▴^	All cancer	Age, alcohol consumption, physical exercise, multivitamin use, menopausal status and postmenopausal hormone use, physical exam in the past 2 years, healthy eating score (quintiles), smoking status, pack-years; BMI, and husband's education	Questionnaire
Hammer et al. (59)	Germany	CS	27,828	NA	337	NA	HR = 0.93^Δ^^▴^	Prostatic cancer	Age and professional status	Questionnaire
Lin et al. (60)	Japan	CS	22,224	52.00	165	NA	NSW: HR = 0.86^Δ^^▴^ Rotating NSW: HR = 1.50^Δ^^▴^	Biliary tract cancer	Age, BMI, history of cholelithiasis, history of diabetes, cigarette smoking, alcohol drinking, perceived stress, and sleep time	Questionnaire
Akerstedt et al. (61)	Sweden	CS	13,656	51.50	463	NA	HR = 0.96^Δ^^▴^	Breast cancer	Age, education level, tobacco consumption, BMI, having children, coffee consumption, previous cancer, use of hormones including oral contraceptives Physical activity Alcohol consumption	Interview
Li et al. (62)	China	NCC	6,489	53.40	1,709	Textile workers	>0–12.8 y: HR = 0.99^Δ^^▴^ >12.8–19.92 y: HR = 0.97^Δ^^▴^ >19.92–27.67 y: HR = 0.90^Δ^^▴^ >27.67 y: HR = 0.88^Δ^^▴^	Breast cancer	Age at the beginning of follow-up	Factory records and in-person interviews
Papantoniou et al.(63)	Spanish	CC	3,486	57.37	1,708	NA	OR = 1.18^Δ^^▴^	Breast cancer	Age, center, educational level, parity, menopausal status, family history of breast cancer, BMI, smoking status, oral contraceptive use, leisure time physical activity, alcohol consumption, and sleep duration	Interview
Santi et al. (64)	Canada	CC	1,519	58.00	744	Nurses	OR = 1.39^Δ^^▴^	Breast cancer	Age, family history, level of education, oral contraception use, alcohol consumption, number of births, and age of first menstruation	Questionnaire
Wang et al. (65)	China	CC	1,454	47.50	712	NA	OR = 1.34^Δ^^▴^	Breast cancer	Age, education, BMI, age at menarche, menopausal status, parity, physical activity, breast-feeding, family history of breast cancer, and other sleep factors (24-h sleep duration, night-shift work, or daytime napping)	Interviews
Travis et al. (16)	UK	CS	795,850	65.28	7,710	NA	Million Women Study: RR = 1.00^Δ^^▴^ EPIC-Oxford: RR = 1.07^Δ^^▴^ UK Biobank: RR = 0.78^Δ^^▴^	Breast cancer	Socioeconomic status, parity and age at first birth, BMI, alcohol intake, strenuous physical activity, family history of breast cancer, age at menarche, oral contraceptive use, smoking, living, with a partner, and use of menopausal hormone therapy	Database
Heckman et al. (66)	US	CS	74,323	46.67	212	Nurses	<2 y: HR = 0.85^Δ^^▴^ 2–5.9 y: HR = 0.84^Δ^^▴^ 6–9.9 y: HR = 1.13^Δ^^▴^ ≥10 y: HR = 0.95^Δ^^▴^	Skin cancer	Years of shift work, hours of sleep, sleep adequacy, sleepy days per week, snoring, restless legs syndrome, family history of melanoma, hours spent in sun, number of severe sunburns, sunburn severity, artificial tanning frequency, annual UV at residence, moles on lower legs, natural hair color in adolescence, marital status, financial status, BMI, physical activity, smoking status, menopausal status, hormone use, and healthy eating index	Questionnaire
Dickerman et al. (67)	Swizerland	CS	11,370	40	602	NA	HR = 0.5^Δ^^▴^ Rotating NSW: HR = 1.0^▴^	Prostatic cancer	Age, education, BMI, physical activity, social class, smoking status, alcohol use, snoring, and zygosity	Questionnaire
Gyarmati et al. (68)	Spain	CC	2,855	62.70	374	NA	OR = 1.10^Δ^^▴^	Stomach cancer	Age, sex, educational level, cent re, BMI, cigarette smoking status, family history, and physical activity level	Interviews
Bai et al. (69)	China	CS	25,377	62.72	1,251	NA	0.1–9.9 y: HR = 1.19^Δ^^▴^ 10–19.9 y: HR = 1.06^Δ^^▴^ ≥20 y: HR = 1.08^Δ^^▴^	All cancer	Age, BMI, family history of cancer, alcohol drinking and smoking status, number of children, menopausal status,	Questionnaire
									hormone replacement therapy, and contraception status	
Costas et al. (70)	Spain	CC	2,049	72.00	321	NA	OR = 1.06^Δ^^▴^	Leukemic cancer	Adjusted for region, age, sex, worked on a farm, family history of hematologic malignancies, body mass index, tobacco consumption, sleep problems, and education	Interview
Wegrzyn et al. (71)	US	CS	193,075	54.72	9,159	Nurses	NHS 1–14 y: HR = 1.01^Δ^^▴^ 15–29 y: HR =1 .06^Δ^^▴^ ≥30 y: HR = 0.95^Δ^^▴^ NHS2 1–9 y: HR = 1.04^Δ^^▴^ 10–19 y: HR = 0.94^Δ^^▴^ ≥20 y: HR = 1.40^Δ^^▴^	Breast cancer	Age, height, BMI, adolescent body size, age at menarche, age at first birth and parity combined, breast feeding, type of menopause and age duration mammography use activity, and current alcohol consumption, physical history of benign breast disease, family history of breast cancer, hormone therapy, first-degree progesterone menopausal duration of estrogen and hormone therapy	Database
Vistisen et al. (23)	Denmark	CS	155,540	39.40	1,245	Nurses	RR = 0.90^Δ^^▴^	Breast cancer	Calendar year, age, age at birth of first child, number of births, family history of breast cancer or ovarian cancer, oral contraception, hormone replacement therapy, other sex hormones, medication, mammography screening attendance, and highest family educational level	Database
Jorgensen et al. (72)	Denmark	CS	28,731	44.00	945	Nurses	HR = 1.05^Δ^^▴^ Rotating NSW: HR = 0.91^▴^	All cancer	Age, smoking, pack-years, physical activity, BMI, alcohol consumption, diet (vegetables, fruit and fatty meat consumption), pre-existing diseases, health, stressful work environment, marital status, and female reproductive factors	Interviews self-reported
Akerstedt et al. (24)	Sweden	CS	12,322	51.50	454	NA	HR = 0.91^Δ^^▴^	Prostatic cancer	Age, education level, tobacco consumption, BMI, having children, coffee consumption, previous cancer, BMI, body mass index.	Telephone interview
Behrens et al. (25)	Germany	CS	1,757	66.80	76	NA	HR = 2.18^Δ^^▴^	Prostatic cancer	Age at event and adjusted for smoking (never, former smoker, current smoker) and family history of prostate cancer	Questionnaire
Tse et al. (73)	China	CC	833	68.82	431	NA	OR = 1.76^Δ^^▴^	Prostatic cancer	Age at interview, marital status, unemployment status, family prostate cancer history, consumption of deeply fried food, consumption of pickled vegetable, and green tea drinking habits	Questionnaire
Papantoniou et al. (20)	US	CS	190,810	42.87	1,965	Nurses	NHS 1–14 y: RR = 1.04^Δ^^▴^ ≥15 y: RR = 1.15^Δ^^▴^	Colorectal cancer	Age, height, BMI, educational level, menopausal status, menopausal hormone	Questionnaires
								NHS2 1–14 y: RR = 0.81^Δ^^▴^ ≥15 y: RR = 0.96^Δ^^▴^	therapy, first-degree family history of colorectal cancer, alcohol consumption, physical activity, smoking status, and medication	
Wendeu-Foyet et al. (21)	France	CC	1,693	NA	818	NA	OR = 0.97^Δ^^▴^ Fixed NSW: OR = 1.04^▴^ Rotating NSW: OR = 0.81^▴^	Prostatic cancer	Adjusted for age, family history of prostate cancer, race, and education level	Self-report
Walasa et al. (22)	Australia	CC	760	NA	350	NA	OR = 1.06^Δ^^▴^	Colorectal cancer	Potential demographic, lifestyle, and medical confounders	Questionnaire
Jones et al. (74)	UK	CS	102,869	45	2,059	NA	HR = 1.00^Δ^^▴^	Breast cancer	Age, time since recruitment, birth cohort, benign breast disease, family history of breast cancer in 1st degree relatives, socio-economic score, birth weight, height, BMI, age at first pregnancy, parity, breast-feeding, current oral contraceptive use before menopause, alcohol consumption, age started smoking, physical activity, etc.	Questionnaire
Leung et al. (19)	Canada	CC	1,402	NA	496	NA	<5.5 y: OR = 1.07^Δ^^▴^ ≥5.5 y: OR = 0.88^Δ^^▴^	Ovarian cancer	Adjusted for age (continuous), education (< high school, high school, college/technical, University undergraduate, University graduate) and parity (nulliparous, 1, 2, ≥3 full-term births)	Interview

BMI, body mass index; CC, case-control study; CS, cohort study; HR, hazard ratio; N, number; NA, not available; NCC, nest case-control study; NSW, night-shift work; OR, odds ratio; RR, relative risk; NHS, nurses' health study;

Δ data included in overall meta-analysis;

▴ *data included in subgroup meta-analysis*.

The quality assessment of original studies showed that no study had an overall low risk of bias and 44 studies were of moderate risk [(4, 16, 19–21, 23–26, 29, 31, 32, 36, 37, 39–43, 46, 48–53, 57–60, 62, 63, 65–71, 73–75); Table S2]. Thirty-one studies were considered to have a low risk of bias in how they defined night-shift work (4, 20, 21, 23, 24, 26, 29, 32, 35–37, 41, 43, 46, 48, 50–53, 58–60, 63, 65, 67–72, 74). For method of exposure measurement, only eight studies showed low risk (20, 23, 36, 41, 57–59, 69). Thirty-one studies had a low risk of bias for reliability of exposure assessment (4, 16, 19–21, 23, 29–31, 33, 35, 37–39, 41, 45, 48, 51, 55, 57–61, 65, 66, 68–70, 73, 75). Forty studies had low risk of bias for the analysis domain (4, 16, 19, 20, 22, 23, 26, 29, 31, 35–37, 39–42, 44, 46–52, 55, 57, 59, 61–74), and 49 reported a low risk in adjustment for confounding factors (4, 16, 20, 21, 23–26, 29, 32, 35–43, 45–55, 57–75). For the aspect of attrition domain, 27 studies had low risk in bias (4, 19, 23–26, 31–33, 37, 40–43, 46, 48, 50, 52, 56–58, 60, 61, 65, 66, 72, 74, 75). Nineteen studies were considered a low risk of bias for blinding (19, 29, 31, 32, 34, 37–40, 42, 43, 47, 49, 50, 58, 62, 63, 69, 70, 75). Fifty-two studies had a low risk in the aspect of selective reporting (4, 16, 19–24, 26, 29–45, 48–54, 56–75). Forty-six studies reported that sponsors had no role in conduct (4, 16, 19–26, 26, 29, 31–36, 39–41, 43, 45–49, 51–63, 65–75) and authors from 51 articles confirmed no conflict of interest (4, 16, 19–26, 29, 31–75).

### Quantitative Meta-Analyses

As shown in Table 2, the risks of breast cancer (pooled *OR* = 1.009, 95% CI = 0.984–1.033), prostate cancer (pooled *OR* = 1.027, 95% CI = 0.982–1.071), ovarian cancer (pooled *OR* = 1.027, 95% CI = 0.942–1.113), pancreatic cancer (pooled *OR* = 1.007, 95% CI = 0.910–1.104), colorectal cancer (pooled *OR* = 1.016, 95% CI = 0.964–1.068) were not significantly associated with night-shift work. Besides, the pooled results, which were from small number of original studies, showed that stomach cancer, esophageal cancer, leukemia, oral cancer, uterine cancer, laryngeal cancer, testicular cancer, and non-Hodgkin lymphoma were not associated with night-shift work. Combination of three original studies showed that night-shift work increased the risk of urinary cancer. However, decreased risks of lung cancer and skin cancer were observed on the basis of pooled results of six and four studies, respectively.

**Table 2:** Meta-analyses on the associations between night-shift work and each type of cancers.

**Types of cancers**	***N***	**OR**	**95% CI**	**Heterogeneity (*I*^**2**^, %)**
Breast cancer	26	1.009	(0.984, 1.033)	45.7
Prostate cancer	12	1.027	(0.982, 1.071)	52.3
Ovarian cancer	8	1.027	(0.942, 1.113)	33.3
Pancreatic cancer	6	1.007	(0.910, 1.104)	3.2
Colorectal cancer	6	1.016	(0.964, 1.068)	52.2
Lung cancer	6	0.949	(0.903, 0.996)	52
Non-Hodgkin's lymph	5	1.046	(0.994, 1.098)	30.5
Stomach cancer	4	1.064	(0.971, 1.157)	0
Skin cancer	4	0.916	(0.879, 0.953)	29.4
Urinary organs	3	1.091	(1.019, 1.163)	0
Esophagus cancer	3	0.812	(0.616, 1.008)	32.1
Leukemia	3	0.983	(0.838, 1.127)	0
Uterus cancer	2	0.984	(0.844, 1.123)	31.9
Oral cancer	2	0.897	(0.717, 1.077)	0
Larynx cancer	2	1.003	(0.784, 1.222)	0
Testis cancer	2	0.9	(0.636, 1.164)	0

*OR, odds ratio; CI, confidence interval; N, number of included studies*.

### Subgroup Analysis

As subgroup analyses on the association between night-shift work and combined risk for cancers (Table 3), no significant associations were observed in cohort studies (pooled *OR* = 0.996, 95% CI = 0.982–1.011) and nest case-control studies (pooled *OR* = 0.960, 95% CI = 0.887–1.032). However, the pooled result from case-control studies was statistically significant (pooled *OR* = 1.176, 95% CI =1.122–1.230). With regard to sex, night-shift work was only associated with increased risk of cancer among men (pooled *OR* = 1.033, 95% CI = 1.010–1.055).

**Table 3:** Subgroup meta-analyses on the associations between night-shift work and combined risk for cancers.

**Groups**	***N***	**OR**	**95% CI**	**Heterogeneity (*I*^**2**^, %)**
**Types of night-shift work**				
Fixed	8	0.880	(0.746, 1.014)	0.0
Rotating	15	0.982	(0.960, 1.004)	41.6
**Study designs**				
Cohort studies	32	0.996	(0982, 1.011)	41.2
Case-control studies	21	1.176	(1.122, 1.230)	47.2
Nest case-control studies	4	0.960	(0.887, 1.032)	18.1
**Sex**				
Women	41	0.989	(0.972, 1.006)	46.7
Men	17	1.033	(1.010, 1.055)	53.1
**Occupation**				
Nurses	11	0.979	(0.958, 1.001)	40.8
Textile workers	2	0.885	(0.826, 0.944)	0.0
**Region**				
Asia	13	0.949	(0.899, 0.998)	39.4
Europe	26	1.019	(1.000, 1.037)	41.3
North America	16	0.999	(0.978, 1.021)	59.1
Oceania	2	1.140	(0.957, 1.323)	0.0

*OR, odds ratio; CI, confidence interval; N, number of included studies*.

For types of night-shift work, neither rotating nor fixed night-shift work was associated with an increased risk of cancer, with pooled ORs 0.982 (95% CI = 0.960–1.004), and 0.880 (95% CI = 0.746–1.014), respectively. The association between night-shift work and cancer risks were also analyzed among occupational groups. Night-shift work was associated with a decreased risk of cancers among textile workers, while no significant association was found for nurses.

We included 13 original studies from Asia, 26 from Europe, 16 from North America, and 2 from Oceania. Subgroup analyses indicated that night-shift work was associated with an increased risk for cancers in Europe, and a decreased risk in Asia, while no significant associations were observed in America or Oceania.

### Dose Response Meta-Analysis

**Figure 3:**
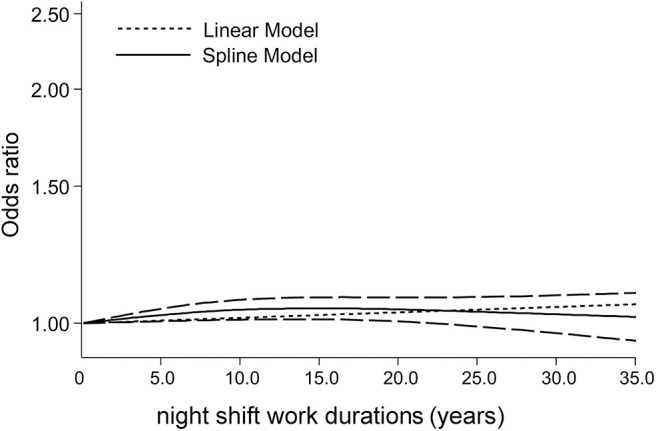
Dose-response analysis on night-shift work durations and cancer risk.

For studies that reported more than one category of durations of night-shift work, we assessed whether the risk of cancer increased in a dose-response manner per year night-shift work. As shown in Figure 3, a dose-response curve was established, in which the solid line is a curve model established by the dose response meta-analysis, while the dotted line represents a reference of linear model. The result indicated that, for every 1 year increase of night-shift work, there is no increased risk for cancer (χ^2^ = 3.34, *P* = 0.067).

**Figure 4:**
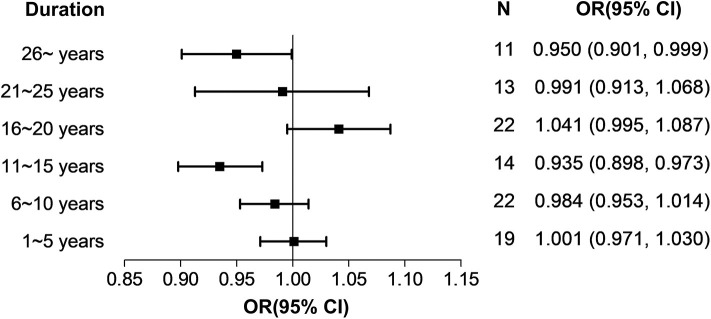
Analysis on classifications of night-shift work duration.

In addition, we validated this finding with comparisons of cancer risks among individuals with different classifications of night work duration (0–5, 6–10, 11–15, 16–20, 21–25, and ≥26 years). Taking all eligible studies together, night-shift work did not increase the risk of cancer in any group of night workers (Figure 4 and Table S3).

### Heterogeneity Analysis

The *Q*-test and *I*^2^ statistics were used to assess heterogeneity across included studies. No obvious heterogeneity was observed in the overall analyses, while heterogeneity was found in four subgroup analyses (i.e., men, North America area, prostate cancer, lung cancer, and colorectal cancer).

### Sensitivity Analysis and Publication Bias

We performed the sensitivity analysis by omitting one study at a time, and recalculated the pooled OR of the remaining studies. As shown in Figure S1, no significant alteration was observed by removal of any single study except when the study of Poole et al. was removed (pooled *OR* = 1.020, 95% CI = 1.000–1.050) (40).

**Figure 5:**
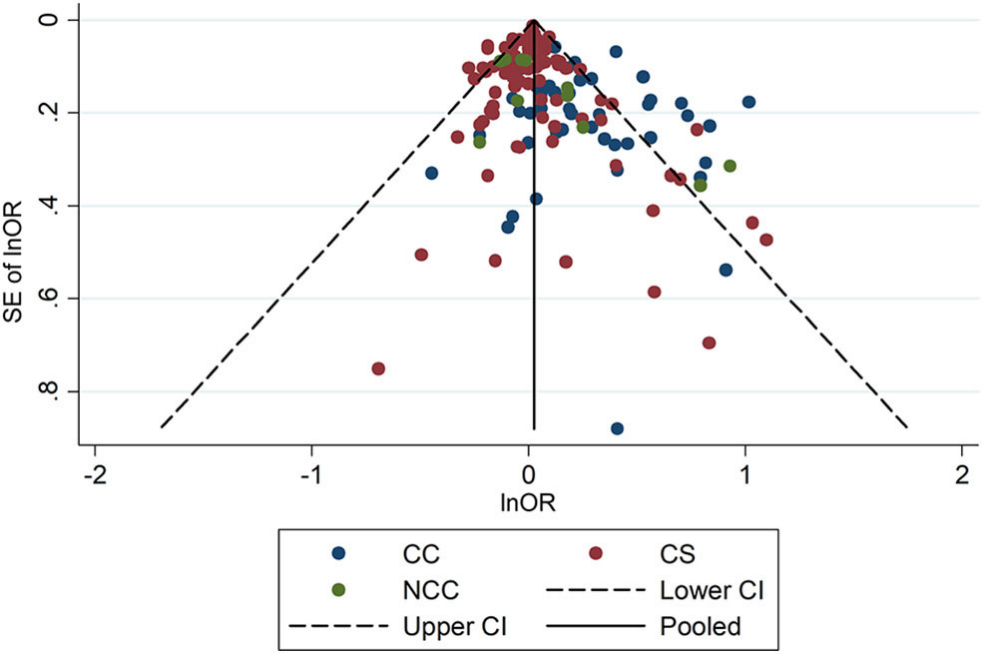
Funnel plot for publication bias analysis.

The Begg's test and funnel plot analysis (Figure 5) revealed a significant publication bias (*z* = 3.45, *P* = 0.001). Complete details in the underlying case-control studies that manifested insignificant associations might not have been fully published. In order to eliminate the fallacy introduced by publication bias, we performed a trim and fill analysis, in which the negative insignificant studies were filled (Figure S2). The filled results showed that night-shift work was not associated with breast cancer (Filled *OR* = 1.018, 95% CI = 0.965–1.074), prostate cancer (Filled *OR* = 1.066, 95% CI = 0.906–1.254); pancreatic cancer (Filled *OR* = 1.051, 95% CI = 0.919–1.203), ovarian cancer (Filled *OR* = 1.050, 95% CI = 0.948–1.164), lung cancer (Filled *OR* = 0.957, 95% CI = 0.861–1.065), or colorectal cancer (Filled *OR* = 1.091, 95% CI = 0.976–1.220). Meanwhile, the trim and fill analysis showed a significant association between night-shift work and skin cancer (Filled *OR* = 0.928, 95% CI = 0.875–0.985). In addition, trim and fill analysis showed insignificant results in Asians (Filled *OR* = 1.002, 95% CI = 0.906–1.108) and Americans (Filled *OR* = 1.016, 95% CI = 0.961–1.075), and a significant result among Europeans (Filled *OR* = 1.058, 95% CI = 1.011–1.106).

## Discussion

This updated systematic review included 57 publications with 8,477,849 participants. Our meta-analysis found an insignificant association between night-shift work and cancer risks. No increased risk for cancer was identified among female night-shift workers as well. Neither rotating night-shift workers nor fixed night-shift workers had an increased risk for cancer. However, analysis on geographical distribution showed an increased risk for cancer among night-shift workers in Europe.

As a common concern in case-control studies, recall bias might have been introduced into our study during the measurement of night work. This bias represents a major threat to the validity when the participants were investigated with self-reported questionnaires. In order to eliminate potential recall bias resulting from previous case-control studies on the association between night-shift work and cancer risks, we synthesized the data from cohort studies in which recall bias can be effectively controlled. Consequently, an insignificant association was noted again.

Researchers have proposed several underlying mechanism of cancer risks induced by night-shift work. Night-shift workers usually experience unnatural light at night, which reduces the release of melatonin (76). As a kind of methoxy indole compound secreted by the pineal gland, melatonin shows a variety of anti-tumor effects, such as anti-oxidant, anti-apoptosis, anti-angiogenesis, as well as modulation of hormones and immunity (77, 78). It has been demonstrated that melatonin plays critical roles in breast, ovarian, endometrial, prostate, lung, and gastrointestinal cancers (79–86). Decreased melatonin leads to an imbalance of inflammatory cytokine secretions, mutagenesis, and oxidative damage, which likely results in the progression of various cancers (87). Suppression of melatonin also induces the aberrant secretion of testosterone and estrogen which increases the risks of prostate, endometrial, ovarian, uterine, and breast cancers (88).

In addition, tumor suppression is a clock-controlled process. Night-shift workers are exposed to dysfunction of circadian genes that is understood to play a role in DNA repair and carcinogen metabolism (89–91). The disruption of the circadian time organization contributes to cancer development. The “clock” genes are known to be directly involved in the regulation of prostate tumorigenesis.

The intensity and duration, as well as the type of night-shift work may influence the effect on cancer risk. As other published systematic reviews, our study included all eligible studies on night-shift work (i.e., fixed and rotating night-shift work) in retrospective and prospective studies. Our subgroup analyses showed that neither fixed nor rotating night-shift work is associated with cancer risk. In addition, night-shift work has little association with cancer risk in spite of the variation of night work duration.

Surprisingly, our subgroup analysis demonstrated that night-shift work is associated with a reduction of cancer risk in Asians. It has been explained that Asian workers have different lifestyles and genotypes compared with Europeans and Americans (2). This finding, as well as the negative association between night-shift work and lung and skin cancers might result from publication bias or relatively small number of included studies.

The present study has more strengths than previous systematic reviews and meta-analyses on the same topic. As a newly released update, this study included many more eligible articles, among them nine studies were that were included in a meta-analysis for the first time. The larger populations enrolled in these studies could produce more accurate effect size at a higher statistical power. Furthermore, our study was conducted on the basis of strict inclusion and exclusion criteria. We rigorously included original data on night-shift work and excluded ineligible studies included in previous meta-analyses. These studies were conducted on work classifications, duration of sleep, sleep disturbance, and light at night. We checked all the database of original studies on night-shift work, and removed three studies on colorectal cancer (58, 92, 93), one study on lung cancer (94), one study on ovarian cancer (40), and five studies on breast cancer (3, 58, 95–97) because these studies reported overlapping data from the Nurses' Health Study (NHS) and/or NHS2. As a preferred solution, the newly published studies were included in our meta-analysis (20, 58).

### Limitations

Our study has some limitations which might sometimes exist in common systematic reviews. First, even though we searched three most commonly used databases, there is potential studies that were missed, especially published in local languages. A slightly different search by a reviewer can lead to very different initial results, which should also cause some caution. Second, we observed moderate heterogeneity in the subgroup analyses of the cohort study group, case-control study group, women, European region, Asian region, breast cancer, lung cancer, and endocrine cancers. The data from case-control studies might be biased by different methods of night-shift work measurement. Third, due to the lack of information on occupations of participants and measurement of night-shift work, these variables were not taken into account in the adjustment model. Moreover, publication bias was statistically positive, which could hinder the quality of this study. We use a trim and fill approach, and no substantial differences were obtained. Our combination of the results on all type of cancers may lead to the neglect of cancer-specific differences. As is known, cancers with stronger hormone components appear to be substantially different from those with less hormone control. In addition, we reported the results for ever vs. never night-shift work. There are many other indicators in night-shift work studies that track exposure in a more variable way. It is possible that the use of more differentiated exposure metrics, such as frequency or intensity of night-shift work, might lead to other results.

In conclusion, this systematic review of 57 observational studies did not find an overall association between ever-exposure to night-shift work and the risk of breast, prostate ovarian, pancreatic, colorectal, non-Hodgkin's lymph, and stomach cancers. With regard to sex, night-shift work was only associated with increased risk of cancer among men.

## Data Availability Statement

Publicly available datasets were analyzed in this study. This data can be found here: https://pubmed.ncbi.nlm.nih.gov/, https://www.embase.com/, and http://isiknowledge.com.

## Author Contributions

HH and YW designed this study. AD, XZ, and XG contributed to literature search, review, and data extraction. XZ and TW conduced statistical analyses. XZ, XJ, and HH contributed to manuscript drafting. AD and YW contributed to manuscript revision. All authors have reviewed and approved the final version of this manuscript.

## Conflict of Interest

The authors declare that the research was conducted in the absence of any commercial or financial relationships that could be construed as a potential conflict of interest.
