# Corrigendum: Association Between Night-Shift Work and Cancer Risk: Updated Systematic Review and Meta-Analysis

**DOI:** 10.3389/fonc.2020.01580

**Published:** 2020-09-03

**Authors:** Aishe Dun, Xuan Zhao, Xu Jin, Tao Wei, Xiang Gao, Youxin Wang, Haifeng Hou

**Affiliations:** ^1^School of Basic Medical Science, Shandong First Medical University & Shandong Academy of Medical Sciences, Tai'an, China; ^2^School of Public Health, Shandong First Medical University & Shandong Academy of Medical Sciences, Tai'an, China; ^3^Department of Otolaryngology, Head and Neck Surgery, Beijing Tongren Hospital, Capital Medical University, Beijing, China; ^4^Beijing Key Laboratory of Clinical Epidemiology, School of Public Health, Capital Medical University, Beijing, China

**Keywords:** night-shift work, carcinogenicity, meta-analysis, risk factor, odds ratio

In our recently published article we noticed that we presented incorrect information in Table 1. A lot of original studies included in our meta-analysis reported stratified estimates of the association between night-shift work and cancer risk; however, we only presented one stratification of the estimates for each study in Table 1. In addition, the number of cases in each study was incorrect. Such errors do not exist in our original dataset of meta-analysis and so the pooled results are not influenced. The corrected Table 1 appears below.

**Table 1:** Characteristics of included studies.

**References**	**Country**	**Study design**	***N* of participants**	**Mean age**	***N* of case**	**Occupations**	**Estimates of risk**	**Cancer**	**Covariates adjusted**	**Measurement of night-shift work**
Hansen et al. (29)	Denmark	CC	12,485	NA	6,281	NA	OR = 1.5^Δ^^▴^	Breast cancer	Age, social class, age at birth of first child, age at birth of last child, and number of children	Interview
Davis et al. (30)	US	CC	1,606	NA	767	NA	OR = 1.13^Δ^^▴^	Breast cancer	Parity, family history of breast cancer, oral contraceptive use, and recent is continued use of hormone replacement therapy	Interview
Lie et al. (31)	Norway	NCC	44,835	NA	537	Nurses	0–14 y: OR = 0.95^Δ^^▴^ 15–29 y: OR = 1.29^Δ^^▴^ ≥30 y: OR = 2.21^Δ^^▴^	Breast cancer	Total duration of work as a nurse and parity	Self-report
Kubo et al. (32)	Japan	CS	14,052	52.14	31	NA	Fixed NSW: RR = 2.3^Δ^^▴^ Rotating NSW: RR = 3.0^Δ^^▴^	Prostatic cancer	Age, study area, family history of prostate cancer, body mass index, smoking, alcohol drinking, job type, physical activity at work, workplace, perceived stress, educational level, and marriage status	Questionnaire
O'Leary et al. (33)	US	CC	1,161	57.19	835	NA	OR = 1.04^Δ^^▴^	Breast cancer	Age at reference date, parity, family history, education, and history of benign breast disease.	In-house interview
Schwartzbaum et al. (34)	Sweden	CS	3,250,787	NA	300,771	NA	Female: OR = 1.00^Δ^^▴^ Male: OR = 1.02^Δ^^▴^	All cancer	Age, socioeconomic status, occupational position, and county of residence of residence	Personal interviews
Viswanathan et al. (4)	US	CS	53,487	53.51	515	Nurses	0–9 y: RR = 0.89^Δ^^▴^ 10–19 y: RR = 1.06^Δ^^▴^ ≥20 y: RR = 1.47^Δ^^▴^	Uterus cancer	Age, age at menarche, age at menopause, parity, BMI, oral contraceptive use, use and duration of postmenopausal hormones, hypertension, diabetes, and smoking	Questionnaire
Marino et al. (35)	US	CC	2,125	NA	812	NA	OR = 1.2^Δ^^▴^	Ovarian cancer	Multivariable adjustment	Interviews
Lahti et al. (36)	Finland	CS	1,669,272	NA	3,813	NA	RR = 1.10^Δ^^▴^	Non-Hodgkin's lymphoma	Age, social class, and cohort period	Questionnaire
Pronk et al. (37)	China	CS	73,049	52.50	717	NA	0–14 y: HR = 1.1^Δ^^▴^ 15–25 y: HR = 0.9^Δ^^▴^ >25 y: HR = 1.0^Δ^^▴^	Breast cancer	Age, education, family history of breast cancer, number of pregnancies, age at first birth, and occupational physical activity	Interview
Chu et al. (38)	China	NCC	2,023	NA	408	NA	HR = 2.54^Δ^^▴^	Breast cancer	Potential cofounders	Interview
Pesch et al. (39)	Germany	CC	1,749	NA	857	NA	>0–4 y: OR = 0.64^Δ^^▴^ 5–9 y: OR = 0.93^Δ^^▴^ 10–19 y: OR = 0.91^Δ^^▴^ ≥20 y: OR = 2.49^Δ^^▴^	Breast cancer	A potential selection bias using bootstrapping, family history of breast cancer, hormone replacement use, and number of mammograms	Interview
Poole et al. (40)	US	CS	181,548	57.21	718	Nurses	1–2 y: HR = 1.07^Δ^^▴^ 3–5 y: HR = 0.90^Δ^^▴^ 6–9 y: HR = 0.92^Δ^^▴^ 10–14 y: HR = 1.14^Δ^^▴^ 15–19 y: HR = 1.28^Δ^^▴^ ≥20 y: HR = 0.80^Δ^^▴^	Ovarian cancer	Age, duration of oral contraceptive use, parity, BMI, smoking status, tubal ligation history, menopausal status, family history of ovarian cancer, duration of breast treating, and cohort	Questionnaire
Kubo et al. (41)	Japan	CS	4,995	55.5	17	NA	RR = 1.79^Δ^^▴^	Prostate cancer	Age, body mass index, alcohol intake, smoking, exercise, and marital status	Questionnaire
Lie et al. (26)	Norway	NCC	1,594	54.46	699	Nurses	1–14 y: OR = 1.2^Δ^^▴^ 15–29 y: OR = 1.2^Δ^^▴^ ≥30 y: OR = 0.8^Δ^^▴^	Breast cancer	Age, period of diagnosis, parity, family history of breast cancer in mother or sister (no/yes), and frequency of alcohol consumption at time of diagnosis	Telephone interview
Hansen et al. (29)	Denmark	CS	1,117	NA	141	Women military	OR = 1.4^Δ^^▴^	Breast cancer	Age, hormone replacement therapy, number of childbirths, age at menarche, years of education, occasional sunbathing frequency, tobacco smoking status	Questionnaire
Parent et al. (42)	Canada	CC	3,670	59.18	761	NA	<5 y: OR = 1.93^Δ^^▴^ 5–10 y: OR = 1.51^Δ^^▴^ >10 y: OR = 1.67^Δ^^▴^	Lung cancer	None	Interview
Natti et al. (43)	Finland	CS	3,095	36.68	51	NA	Men: HR = 1.78^Δ^^▴^ Women: HR = 2.82^Δ^^▴^	All cancer	Age, and smoking status, and health- and work-related factors	Interview
Lin et al. (44)	Japan	CS	22,224	52.19	16	Industry	Fixed NSW: RR = 0.61^Δ^^▴^ Rotating NSW: RR = 0.83^Δ^^▴^	Pancreatic cancer	Age, body mass index, history of diabetes, alcohol drinking, cigarette smoking, perceived stress, and sleep time.	Questionnaire
Knutsson et al. (45)	Sweden	CS	4,036	42.31	94	NA	HR = 2.02^Δ^^▴^	Breast cancer	Number of children, alcohol consumption, BMI, height, weight, waist, hip circumference, educational level, smoking menopausal status, status of oral contraceptive use, and hormones other than contraceptives	Questionnaire
Bhatti et al. (46)	US	CC	3,322	NA	1,490	NA	Invasive: OR = 1.24^Δ^ Borderline: OR = 1.48^Δ^	Ovarian cancer	Age at reference, county, reference year, duration of oral contraceptive use, number of full-term pregnancies, and BMI at age 30	Interviews self-reported
Fritschi et al. (47)	Australian	CC	2,987	NA	1,202	NA	OR = 1.16^Δ^^▴^	Breast cancer	Night shift work	Questionnaire
Menegaux et al. (48)	France	CC	2,549	NA	1,232	NA	OR = 1.27^Δ^^▴^	Breast cancer	Age, study area, parity, age at first full term pregnancy, age at menarche, family history of breast cancer, current hormonal replacement therapy, BMI, tobacco, and alcohol	Interview
Grundy et al. (49)	Canada	CC	2,313	57.03	1,134	NA	0–14 y: OR = 0.95^Δ^^▴^ 15–29 y: OR = 0.93^Δ^^▴^ ≥30 y: OR = 2.21^Δ^^▴^	Breast cancer	Years of night shift history	Questionnaire
Rabstein et al. (50)	Germany	CC	1,749	NA	857	NA	OR = 1.01^Δ^^▴^	Breast cancer	Family history of breast cancer, hormone replacement use, number of mammograms, and estrogen receptor status	Interview
Koppes et al. (51)	Netherland	CS	285,723	NA	2,531	Employed women	HR = 0.87^Δ^^▴^	Breast cancer	Age, origin, children in household education, occupation, job tenure (years)	Interview
Gapstur et al. (52)	US	CS	305,057	51.44	4,836	NA	Fixed NSW: RR = 0.72^Δ^^▴^ Rotating NSW: RR = 1.08^Δ^^▴^	Prostate cancer	Age, race, education, BMI, smoking status, family history of prostate cancer, and painful/frequent urination	Questionnaire
Carter et al. (53)	US	CS	161,004	50.28	1,253	Employed women	Fixed NSW: R = 1.12^Δ^^▴^ Rotating NSW: RR = 1.27^Δ^^▴^	Ovarian cancer	Oral contraceptive use, age at menarche and menopause, tubal ligation, parity, postmenopausal estrogen use, race, family history of breast/ovarian cancers, exercise, BMI, and height	Questionnaire
Yong et al. (54)	Germany	CS	27,828	40.05	1,073	Chemical workers	HR = 1.04^Δ^	All cancer	Age, job level, cigarette smoking, and employment duration in categories	Questionnaire
Ren et al. (55)	China	CC	1,454	NA	712	NA	OR = 1.34^Δ^^▴^	Breast cancer	Age, education, BMI, marital status, age at menarche, menopausal status, parity, activity, breastfeeding, family history of breast cancer, and other sleep factors	Database
Datta et al. (56)	India	CC	150	NA	50	NA	OR = 1.51^Δ^^▴^	Breast cancer	Age, obesity factors, and food habits	Interview
Kwon et al. (57)	China	CS	4,471	54.01	1,451	Textile workers	0–17.1 y: HR = 0.76^Δ^^▴^ 17.1–24.9 y: HR = 0.89^Δ^^▴^ 24.9–30.6 y: HR = 0.94^Δ^^▴^ >30.6 y: HR = 0.82^Δ^^▴^	Lung cancer	Age, smoking, parity, and endotoxin	Factory record
Gu et al. (58)	US	CS	71,857	63.98	5,413	Nurses	1-5 y: HR = 1.03^Δ^^▴^ 6–14 y: HR=1.04^Δ^^▴^ ≥15 y: HR = 1.08^Δ^^▴^	All cancer	Age, alcohol consumption, physical exercise, multivitamin use, menopausal status and postmenopausal hormone use, physical exam in the past 2 years, healthy eating score (quintiles), smoking status, pack-years; BMI, and husband's education	Questionnaire
Hammer et al. (59)	Germany	CS	27,828	NA	337	NA	HR = 0.93^Δ^^▴^	Prostatic cancer	Age and professional status	Questionnaire
Lin et al. (60)	Japan	CS	22,224	52.00	165	NA	NSW: HR = 0.86^Δ^^▴^ Rotating NSW: HR = 1.50^Δ^^▴^	Biliary tract cancer	Age, BMI, history of cholelithiasis, history of diabetes, cigarette smoking, alcohol drinking, perceived stress, and sleep time	Questionnaire
Akerstedt et al. (61)	Sweden	CS	13,656	51.50	463	NA	HR = 0.96^Δ^^▴^	Breast cancer	Age, education level, tobacco consumption, BMI, having children, coffee consumption, previous cancer, use of hormones including oral contraceptives Physical activity Alcohol consumption	Interview
Li et al. (62)	China	NCC	6,489	53.40	1,709	Textile workers	>0–12.8 y: HR = 0.99^Δ^^▴^ >12.8–19.92 y: HR = 0.97^Δ^^▴^ >19.92–27.67 y: HR = 0.90^Δ^^▴^ >27.67 y: HR = 0.88^Δ^^▴^	Breast cancer	Age at the beginning of follow-up	Factory records and in-person interviews
Papantoniou et al.(63)	Spanish	CC	3,486	57.37	1,708	NA	OR = 1.18^Δ^^▴^	Breast cancer	Age, center, educational level, parity, menopausal status, family history of breast cancer, BMI, smoking status, oral contraceptive use, leisure time physical activity, alcohol consumption, and sleep duration	Interview
Santi et al. (64)	Canada	CC	1,519	58.00	744	Nurses	OR = 1.39^Δ^^▴^	Breast cancer	Age, family history, level of education, oral contraception use, alcohol consumption, number of births, and age of first menstruation	Questionnaire
Wang et al. (65)	China	CC	1,454	47.50	712	NA	OR = 1.34^Δ^^▴^	Breast cancer	Age, education, BMI, age at menarche, menopausal status, parity, physical activity, breast-feeding, family history of breast cancer, and other sleep factors (24-h sleep duration, night-shift work, or daytime napping)	Interviews
Travis et al. (16)	UK	CS	795,850	65.28	7,710	NA	Million Women Study: RR = 1.00^Δ^^▴^ EPIC-Oxford: RR = 1.07^Δ^^▴^ UK Biobank: RR = 0.78^Δ^^▴^	Breast cancer	Socioeconomic status, parity and age at first birth, BMI, alcohol intake, strenuous physical activity, family history of breast cancer, age at menarche, oral contraceptive use, smoking, living, with a partner, and use of menopausal hormone therapy	Database
Heckman et al. (66)	US	CS	74,323	46.67	212	Nurses	<2 y: HR = 0.85^Δ^^▴^ 2–5.9 y: HR = 0.84^Δ^^▴^ 6–9.9 y: HR = 1.13^Δ^^▴^ ≥10 y: HR = 0.95^Δ^^▴^	Skin cancer	Years of shift work, hours of sleep, sleep adequacy, sleepy days per week, snoring, restless legs syndrome, family history of melanoma, hours spent in sun, number of severe sunburns, sunburn severity, artificial tanning frequency, annual UV at residence, moles on lower legs, natural hair color in adolescence, marital status, financial status, BMI, physical activity, smoking status, menopausal status, hormone use, and healthy eating index	Questionnaire
Dickerman et al. (67)	Swizerland	CS	11,370	40	602	NA	HR = 0.5^Δ^^▴^ Rotating NSW: HR = 1.0^▴^	Prostatic cancer	Age, education, BMI, physical activity, social class, smoking status, alcohol use, snoring, and zygosity	Questionnaire
Gyarmati et al. (68)	Spain	CC	2,855	62.70	374	NA	OR = 1.10^Δ^^▴^	Stomach cancer	Age, sex, educational level, cent re, BMI, cigarette smoking status, family history, and physical activity level	Interviews
Bai et al. (69)	China	CS	25,377	62.72	1,251	NA	0.1–9.9 y: HR = 1.19^Δ^^▴^ 10–19.9 y: HR = 1.06^Δ^^▴^ ≥20 y: HR = 1.08^Δ^^▴^	All cancer	Age, BMI, family history of cancer, alcohol drinking and smoking status, number of children, menopausal status,	Questionnaire
									hormone replacement therapy, and contraception status	
Costas et al. (70)	Spain	CC	2,049	72.00	321	NA	OR = 1.06^Δ^^▴^	Leukemic cancer	Adjusted for region, age, sex, worked on a farm, family history of hematologic malignancies, body mass index, tobacco consumption, sleep problems, and education	Interview
Wegrzyn et al. (71)	US	CS	193,075	54.72	9,159	Nurses	NHS 1–14 y: HR = 1.01^Δ^^▴^ 15–29 y: HR =1 .06^Δ^^▴^ ≥30 y: HR = 0.95^Δ^^▴^ NHS2 1–9 y: HR = 1.04^Δ^^▴^ 10–19 y: HR = 0.94^Δ^^▴^ ≥20 y: HR = 1.40^Δ^^▴^	Breast cancer	Age, height, BMI, adolescent body size, age at menarche, age at first birth and parity combined, breast feeding, type of menopause and age duration mammography use activity, and current alcohol consumption, physical history of benign breast disease, family history of breast cancer, hormone therapy, first-degree progesterone menopausal duration of estrogen and hormone therapy	Database
Vistisen et al. (23)	Denmark	CS	155,540	39.40	1,245	Nurses	RR = 0.90^Δ^^▴^	Breast cancer	Calendar year, age, age at birth of first child, number of births, family history of breast cancer or ovarian cancer, oral contraception, hormone replacement therapy, other sex hormones, medication, mammography screening attendance, and highest family educational level	Database
Jorgensen et al. (72)	Denmark	CS	28,731	44.00	945	Nurses	HR = 1.05^Δ^^▴^ Rotating NSW: HR = 0.91^▴^	All cancer	Age, smoking, pack-years, physical activity, BMI, alcohol consumption, diet (vegetables, fruit and fatty meat consumption), pre-existing diseases, health, stressful work environment, marital status, and female reproductive factors	Interviews self-reported
Akerstedt et al. (24)	Sweden	CS	12,322	51.50	454	NA	HR = 0.91^Δ^^▴^	Prostatic cancer	Age, education level, tobacco consumption, BMI, having children, coffee consumption, previous cancer, BMI, body mass index.	Telephone interview
Behrens et al. (25)	Germany	CS	1,757	66.80	76	NA	HR = 2.18^Δ^^▴^	Prostatic cancer	Age at event and adjusted for smoking (never, former smoker, current smoker) and family history of prostate cancer	Questionnaire
Tse et al. (73)	China	CC	833	68.82	431	NA	OR = 1.76^Δ^^▴^	Prostatic cancer	Age at interview, marital status, unemployment status, family prostate cancer history, consumption of deeply fried food, consumption of pickled vegetable, and green tea drinking habits	Questionnaire
Papantoniou et al. (20)	US	CS	190,810	42.87	1,965	Nurses	NHS 1–14 y: RR = 1.04^Δ^^▴^ ≥15 y: RR = 1.15^Δ^^▴^	Colorectal cancer	Age, height, BMI, educational level, menopausal status, menopausal hormone	Questionnaires
								NHS2 1–14 y: RR = 0.81^Δ^^▴^ ≥15 y: RR = 0.96^Δ^^▴^	therapy, first-degree family history of colorectal cancer, alcohol consumption, physical activity, smoking status, and medication	
Wendeu-Foyet et al. (21)	France	CC	1,693	NA	818	NA	OR = 0.97^Δ^^▴^ Fixed NSW: OR = 1.04^▴^ Rotating NSW: OR = 0.81^▴^	Prostatic cancer	Adjusted for age, family history of prostate cancer, race, and education level	Self-report
Walasa et al. (22)	Australia	CC	760	NA	350	NA	OR = 1.06^Δ^^▴^	Colorectal cancer	Potential demographic, lifestyle, and medical confounders	Questionnaire
Jones et al. (74)	UK	CS	102,869	45	2,059	NA	HR = 1.00^Δ^^▴^	Breast cancer	Age, time since recruitment, birth cohort, benign breast disease, family history of breast cancer in 1st degree relatives, socio-economic score, birth weight, height, BMI, age at first pregnancy, parity, breast-feeding, current oral contraceptive use before menopause, alcohol consumption, age started smoking, physical activity, etc.	Questionnaire
Leung et al. (19)	Canada	CC	1,402	NA	496	NA	<5.5 y: OR = 1.07^Δ^^▴^ ≥5.5 y: OR = 0.88^Δ^^▴^	Ovarian cancer	Adjusted for age (continuous), education (< high school, high school, college/technical, University undergraduate, University graduate) and parity (nulliparous, 1, 2, ≥3 full-term births)	Interview

BMI, body mass index; CC, case-control study; CS, cohort study; HR, hazard ratio; N, number; NA, not available; NCC, nest case-control study; NSW, night-shift work; OR, odds ratio; RR, relative risk; NHS, nurses' health study;

Δ data included in overall meta-analysis;

▴ *data included in subgroup meta-analysis*.

The authors apologize for this error and state that this does not change the scientific conclusions of the article in any way. The original article has been updated.

