# Supplementary material for: Association Between Night-Shift Work and Cancer Risk: Updated Systematic Review and Meta-Analysis
Source: Front Oncol. 2020 Jun 23;10:1006. doi: 10.3389/fonc.2020.01006 (PMC7324664; doi:10.3389/fonc.2020.01006)
Supplement: Supplementary file 1 [file Data_Sheet_1.DOCX]

**[Supplementary materials]**

**Table S1 Checklist of the Preferred Reporting Items for Systematic Reviews and Meta-Analyses (PRISMA)**

| **Section/Topic** | **Checklist Item** | **Reported or not** |
| --- | --- | --- |
| TITLE |  |  |
| Title | Identify the report as a systematic review, meta-analysis, or both. | Y |
| ABSTRACT |  |  |
| Structured summary | Provide a structured summary including, as applicable: background; objectives; data sources; study eligibility criteria, participants, and interventions; study appraisal and synthesis methods; results; limitations; conclusions and implications of key findings; systematic review registration number. | Y |
| INTRODUCTION |  |  |
| Rationale | Describe the rationale for the review in the context of what is already known. | Y |
| Objectives | Provide an explicit statement of questions being addressed with reference to participants, interventions, comparisons, outcomes, and study design (PICOS). | Y |
| METHODS |  |  |
| Protocol and registration | Indicate if a review protocol exists, if and where it can be accessed (e.g., Web address), and, if available, provide registration information including registration number. | Y |
| Eligibility criteria | Specify study characteristics (e.g., PICOS, length of follow-up) and report characteristics (e.g., years considered, language, publication status) used as criteria for eligibility, giving rationale. | Y |
| Information sources | Describe all information sources (e.g., databases with dates of coverage, contact with study authors to identify additional studies) in the search and date last searched. | Y |
| Search | Present full electronic search strategy for at least one database, including any limits used, such that it could be repeated. | Y |
| Study selection | State the process for selecting studies (i.e., screening, eligibility, included in systematic review, and, if applicable, included in the meta-analysis). | Y |
| Data collection process | Describe method of data extraction from reports (e.g., piloted forms, independently, in duplicate) and any processes for obtaining and confirming data from investigators. | Y |
| Data items | List and define all variables for which data were sought (e.g., PICOS, funding sources) and any assumptions and simplifications made. | Y |
| Risk of bias in individual studies | Describe methods used for assessing risk of bias of individual studies (including specification of whether this was done at the study or outcome level), and how this information is to be used in any data synthesis. | Y |
| Summary measures | State the principal summary measures (e.g., risk ratio, difference in means). | Y |
| Synthesis of results | Describe the methods of handling data and combining results of studies, if done, including measures of consistency (e.g., I2) for each meta-analysis. | Y |
| Risk of bias across studies | Specify any assessment of risk of bias that may affect the cumulative evidence (e.g., publication bias, selective reporting within studies). | Y |
| Additional analyses | Describe methods of additional analyses (e.g., sensitivity or subgroup analyses, meta-regression), if done, indicating which were pre-specified. | Y |
| RESULTS |  |  |
| Study selection | Give numbers of studies screened, assessed for eligibility, and included in the review, with reasons for exclusions at each stage, ideally with a flow diagram. | Y |
| Study characteristics | For each study, present characteristics for which data were extracted (e.g., study size, PICOS, follow-up period) and provide the citations. | Y |
| Risk of bias within studies | Present data on risk of bias of each study and, if available, any outcome-level assessment (see Item 12). | Y |
| Results of individual studies | For all outcomes considered (benefits or harms), present, for each study: (a) simple summary data for each intervention group and (b) effect estimates and confidence intervals, ideally with a forest plot. | Y |
| Synthesis of results | Present results of each meta-analysis done, including confidence intervals and measures of consistency. | Y |
| Risk of bias across studies | Present results of any assessment of risk of bias across studies (see Item 15). | Y |
| Additional analysis | Give results of additional analyses, if done (e.g., sensitivity or subgroup analyses, meta-regression [see Item 16]). | Y |
| DISCUSSION |  | Y |
| Summary of evidence | Summarize the main findings including the strength of evidence for each main outcome; consider their relevance to key groups (e.g., health care providers, users, and policy makers). | Y |
| Limitations | Discuss limitations at study and outcome level (e.g., risk of bias), and at review level (e.g., incomplete retrieval of identified research, reporting bias). | Y |
| Conclusions | Provide a general interpretation of the results in the context of other evidence, and implications for future research. | Y |
| FUNDING |  |  |
| Funding | Describe sources of funding for the systematic review and other support (e.g., supply of data) | Y |

Y: the item was reported in article, N: the item was not reported.

**Table S2 Risk of bias of included studies**

| Studies | Exposure  definition | Exposure  assessment | Reliability of exposure  assessment | Analysis/research specific bias | Confounding | Attrition | Blinding of  assessors | Selective  reporting | Funding | Conflict of  interest | Study-level risk of bias |
| --- | --- | --- | --- | --- | --- | --- | --- | --- | --- | --- | --- |
| Hansen, 2001 | HR | HR | LR | HR | LR | LR | LR | LR | LR | LR | Moderate |
| Davis, 2001 | HR | HR | LR | HR | HR | HR | UR | LR | UR | UR | High |
| Lie, 2006 | HR | HR | LR | LR | HR | LR | LR | LR | LR | LR | Moderate |
| Kubo, 2006 | LR | HR | HR | UR | LR | LR | LR | LR | UR | LR | Moderate |
| O’Leary, 2006 | HR | HR | LR | UR | HR | LR | UR | LR | UR | LR | High |
| Schwartzbaum, 2007 | HR | HR | HR | HR | HR | HR | LR | UR | LR | LR | High |
| Viswanathan, 2007 | LR | HR | LR | LR | LR | LR | UR | LR | LR | LR | Moderate |
| Marino, 2008 | LR | HR | LR | LR | LR | HR | UR | LR | LR | LR | Moderate |
| Lahti, 2008 | LR | LR | UR | LR | LR | UR | UR | LR | LR | LR | Moderate |
| Pronk, 2010 | LR | HR | LR | LR | LR | LR | LR | LR | UR | LR | Moderate |
| Chu, 2010 | HR | HR | LR | UR | LR | UR | LR | LR | UR | LR | Moderate |
| Pesch, 2010 | HR | HR | LR | LR | LR | HR | LR | LR | LR | LR | Moderate |
| Poole, 2011 | HR | HR | HR | LR | LR | LR | UR | LR | LR | LR | Moderate |
| Kubo, 2011 | LR | LR | LR | LR | LR | LR | UR | LR | LR | LR | Moderate |
| Lie, 2011 | LR | HR | HR | LR | LR | LR | UR | LR | LR | LR | Moderate |
| Hansen, 2012 | LR | HR | LR | LR | LR | HR | LR | LR | LR | LR | Moderate |
| Parent, 2012 | HR | HR | HR | LR | LR | LR | LR | LR | UR | LR | Moderate |
| Natti, 2012 | LR | HR | HR | UR | LR | LR | LR | LR | LR | LR | Moderate |
| Lin, 2013 | HR | HR | HR | LR | HR | UR | UR | LR | UR | LR | High |
| Knutsson, 2013 | HR | HR | UR | HR | LR | HR | UR | LR | LR | LR | High |
| Bhatti, 2013 | LR | HR | HR | LR | LR | LR | UR | UR | LR | LR | Moderate |
| Fritschi, 2013 | HR | HR | HR | UR | LR | HR | LR | UR | LR | LR | High |
| Menegaux, 2013 | LR | HR | LR | LR | LR | LR | HR | LR | LR | LR | Moderate |
| Grundy, 2013 | HR | HR | HR | LR | LR | HR | LR | LR | LR | LR | Moderate |
| Rabstein, 2013 | LR | HR | UR | LR | LR | LR | LR | LR | UR | LR | Moderate |
| Koppes, 2014 | LR | HR | LR | LR | LR | UR | HR | LR | LR | LR | Moderate |
| Gapstur, 2014 | LR | HR | HR | LR | LR | LR | UR | LR | LR | LR | Moderate |
| Carter, 2014 | LR | HR | UR | HR | LR | UR | UR | LR | LR | LR | Moderate |
| Yong, 2014 | HR | HR | HR | HR | LR | UR | UR | LR | LR | LR | High |
| Ren, 2014 | HR | HR | LR | LR | LR | UR | UR | UR | LR | LR | High |
| Datta, 2014 | HR | HR | UR | UR | UR | LR | HR | LR | LR | LR | High |
| Kwon, 2015 | UR | LR | LR | LR | LR | LR | UR | LR | LR | LR | Moderate |
| Gu, 2015 | LR | LR | LR | UR | LR | LR | LR | LR | UR | LR | Moderate |
| Hammer, 2015 | LR | LR | LR | LR | LR | UR | UR | LR | LR | LR | Moderate |
| Lin, 2015 | LR | HR | LR | HR | LR | LR | UR | LR | LR | LR | Moderate |
| Akerstedt, 2015 | HR | HR | LR | LR | LR | LR | UR | LR | LR | LR | Moderate |
| Li W, 2015 | HR | HR | HR | LR | LR | UR | LR | LR | LR | LR | Moderate |
| Papantoniou, 2015 | LR | LR | LR | LR | LR | UR | UR | LR | LR | LR | Moderate |
| Santi, 2015 | HR | HR | HR | LR | LR | UR | UR | LR | UR | LR | High |
| Wang, 2015 | LR | HR | LR | LR | LR | LR | UR | LR | LR | LR | Moderate |
| Travis, 2016 | HR | HR | LR | LR | LR | UR | UR | LR | LR | LR | Moderate |
| Heckman, 2016 | HR | HR | LR | LR | LR | LR | UR | LR | LR | LR | Moderate |
| Dickerman, 2016 | LR | HR | HR | LR | LR | UR | UR | LR | LR | LR | Moderate |
| Gyarmati, 2016 | LR | HR | LR | LR | LR | UR | UR | LR | LR | LR | Moderate |
| Bai, 2016 | LR | LR | LR | LR | LR | UR | LR | LR | LR | LR | Moderate |
| Costas, 2016 | LR | HR | LR | LR | LR | UR | LR | LR | LR | LR | Moderate |
| Wegrzyn, 2017 | LR | HR | HR | LR | LR | HR | UR | LR | LR | LR | Moderate |
| Vistisen, 2017 | LR | LR | LR | LR | LR | LR | UR | LR | LR | LR | Moderate |
| Jorgensen, 2017 | LR | HR | HR | LR | LR | LR | UR | LR | LR | LR | Moderate |
| Akerstedt, 2017 | LR | HR | HR | HR | LR | HR | UR | LR | UR | LR | High |
| Behrens, 2017 | HR | HR | UR | UR | LR | LR | UR | UR | LR | LR | High |
| Tse, 2017 | HR | HR | LR | LR | LR | HR | UR | LR | LR | LR | Moderate |
| Papantoniou, 2018 | LR | HR | UR | LR | LR | UR | LR | LR | LR | LR | Moderate |
| Wendeu-Foyet, 2018 | LR | HR | LR | UR | LR | HR | UR | LR | LR | LR | Moderate |
| Walasa, 2018 | HR | HR | HR | LR | UR | UR | UR | LR | LR | LR | High |
| Jones, 2019 | LR | HR | UR | LR | LR | LR | UR | LR | LR | LR | Moderate |
| Leung, 2019 | HR | HR | LR | LR | UR | LR | LR | LR | LR | LR | Moderate |

HR: high risk of bias; LR: low risk of bias; UR: unclear risk of bias

**Table S3 Analyses on classifications of night shift work durations**

| **Cancer** | **Durations (years)** | **N** | **OR** | **95% CI** | **Heterogeneity (I^2^, %)** |
| --- | --- | --- | --- | --- | --- |
| All cancer risks | 1-5 | 19 | 1.001 | (0.971, 1.030) | 54.7 |
|  | 6-10 | 22 | 0.984 | (0.953, 1.014) | 26.7 |
|  | 11-15 | 14 | 0.935 | (0.898, 0.973) | 42.7 |
|  | 16-20 | 22 | 1.041 | (0.995, 1.087) | 0.0 |
|  | 21-25 | 13 | 0.991 | (0.913, 1.068) | 44.5 |
|  | ≥26 | 11 | 0.950 | (0.901, 0.999) | 8.7 |
| Breast cancer risk | 1-5 | 10 | 0.984 | (0.778, 1.190) | 73.3 |
|  | 6-10 | 17 | 1.058 | (0.978, 1.139) | 61.6 |
|  | 11-15 | 3 | 0.889 | (0.659, 1.218) | 83.8 |
|  | 16-20 | 11 | 1.017 | (0.938, 1.096) | 0.0 |
|  | 21-25 | 13 | 0.989 | (0.909, 1.068) | 8.0 |
|  | ≥26 | 9 | 0.973 | (0.829, 1.116) | 17.7 |

OR, odds ratio; CI, confidence interval; N, number of included studies


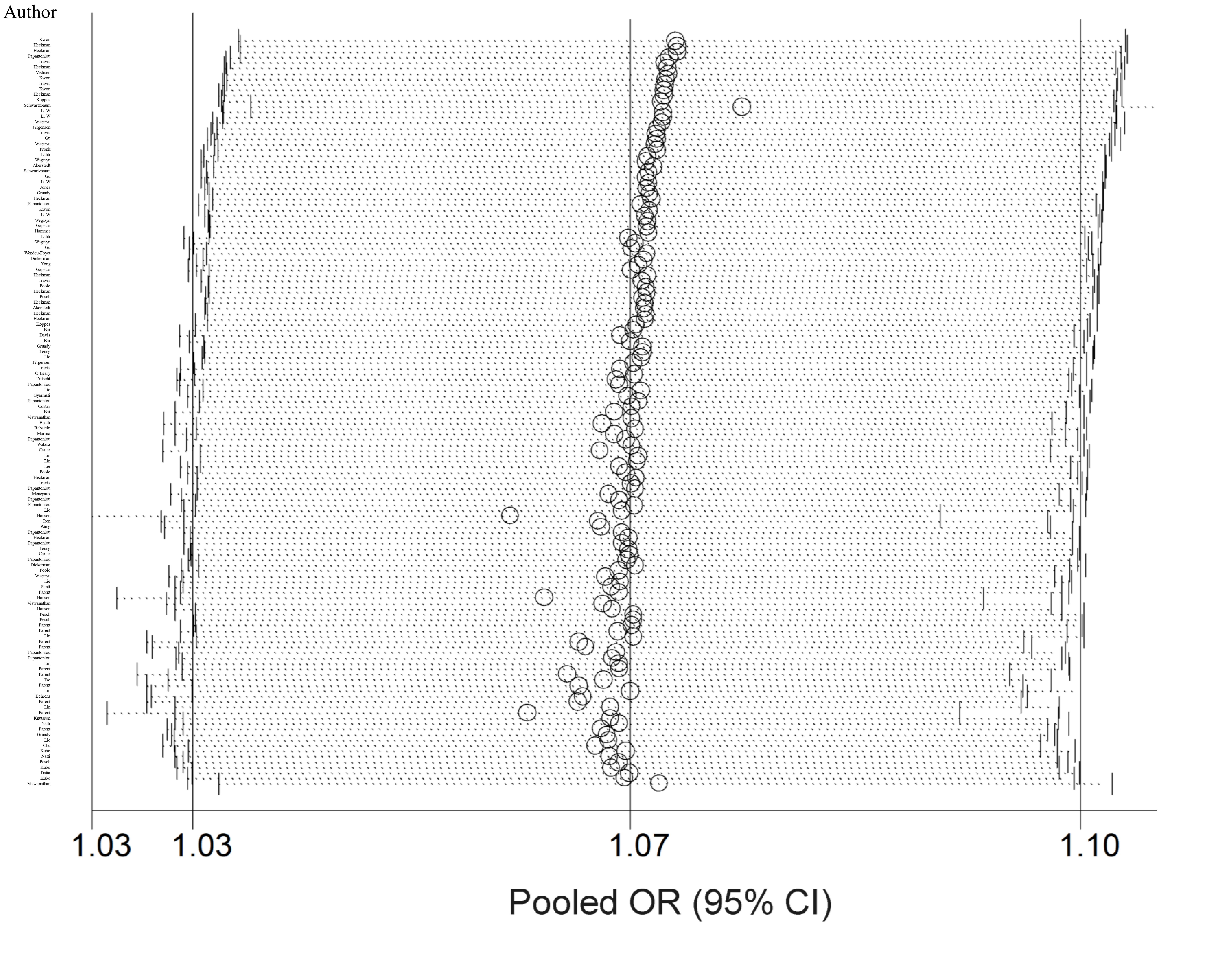


**Figure S1 Sensitivity analysis on removal of each studies**

OR, odds ratio; CI, confidence interval; Each dot line is the OR and 95% CI of the study removed for sensitivity analysis


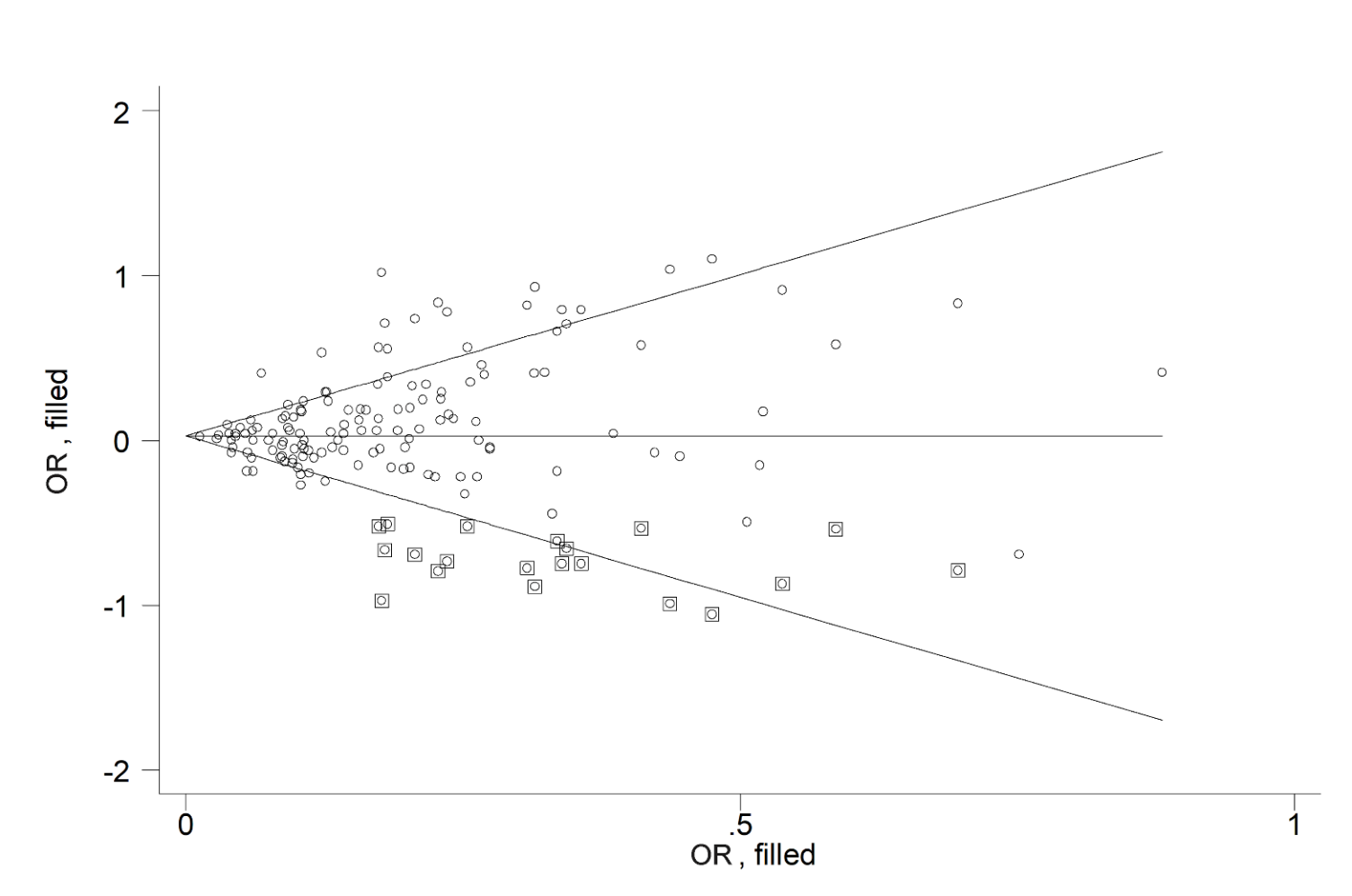


**Figure S2 Trim and fill analysis for control of publication bias**

OR, odds ratio; CI, confidence interval; Each circle represents one original study; The squares represent the filled studies;he OR and 95% CI of the study removed for sensitivity analysis

**Supplementary Box 1 Literature search process**

| 5) #4 AND English[Language]  4) #3 AND published up to 2019/05/31  3) #1 AND #2  2) carcinoma[Title/Abstract] OR tumor[Title/Abstract] OR cancer[Title/Abstract] OR neoplasm[Title/Abstract]  1) night shift work[Title/Abstract] OR shift work[Title/Abstract] OR night work[Title/Abstract] |
| --- |

**Supplementary Box 2 Assessment of risk of bias**

| **Exposure definition**  Low risk: The definition included at least two of the following three aspects: shift system (rotating or fixed, forward or backward rotation); shift duration (in years); and shift intensity (per week or per month frequency).  High risk: A categorical definition with an arbitrary threshold or a definition that covers only one aspect of exposure (start or end time of shift or duration, intensity, or shift system).  Unclear: No definition.  **Assessment of exposure**  Low risk: The exposure was objectively measured such as direct measurement of exposure, such as logging data, shift schedule data from the human resources or employers’ records, and prospective self-measurement of exposure, e.g., diaries  High risk: The exposure was assessed using subjective measures: reported by participants (interviews/questionnaires) or a proxy used to allocate exposure status (job matrix, job title).  Unclear: Not available.  **Blinding**  Low risk: Assessors were reported to be blind to assess exposure status and case status.  High risk: Assessors were not blind to exposure or case status.  Unclear: A lack of information led to a judgment of unclear.  **Reliability of exposure estimates**  Low risk: When good inter/intra observer reliability was achieved with reported reliability values or when objective measures were used (such as log data), cohort studies’ reliability of exposure estimates were judged to have a low risk of bias.  High risk: Observer variability was reported by means of a subjective judgment of reliability.  Unclear: A lack of information led to a judgment of unclear.  **Confounder assessment**  Low risk: Major confounding factors/effect modifiers (*e.g.* age, BMI, ethnicity, and socioeconomic status) were assessed completely.  High risk: Major confounding factors assessed partially.  **Attrition**  Low risk: A total loss of participants (non-response in case–control studies) of <20% loss in total, and ≤10% difference in dropout/non-response between the two groups.  High risk: A≥20% or a dropout/non-response difference between the compared groups of ≥10% or the reasons for dropout/non-response not given/different led to a judgment of high risk.  Unclear: Lack of information.  **Selective reporting of results**.  Low risk: Adjusted estimates were presented for all hypotheses tested as per aims.  High risk: Incomplete/selective reporting of the tested hypotheses (compared to aim and objectives) and/or crude estimates only.  Unclear: Lack of information, or ambiguous concept.  **Research-specific bias**  Low risk: All three of the following aspects were used: (1) the methods used to reduce bias due to research design (e.g. standardization, matching, adjustment in multivariate model, stratification, and propensity scoring), (2) the assessment of dose–response effect (e.g. subgroup, regression), and (3) author justification of the sample size, in descending order of importance.  High risk: One of these aspects in a particular study.  **Funding**  This was assessed in two areas: source of funding and the involvement of the funding body in the research.  Low risk: A study was funded by non-profit organization(s) and it was clear that the funding body was not involved in the conduct or interpretation of the research.  High risk: One of these factors was high risk, the study was considered to have a high risk of bias.  Unclear: No reported.  **Conflicts of interest**  Low risk: No conflicts of interests.  High risk: One or more authors indicated a conflicting interest.  Unclear: No mentioned. |
| --- |
